# Retinomorphic Motion Detector Fabricated with Organic Infrared Semiconductors

**DOI:** 10.1002/advs.202304688

**Published:** 2023-09-06

**Authors:** Shuo‐En Wu, Longhui Zeng, Yichen Zhai, Chanho Shin, Naresh Eedugurala, Jason D. Azoulay, Tse Nga Ng

**Affiliations:** ^1^ Materials Science and Engineering Program University of California San Diego La Jolla CA 92093 USA; ^2^ Department of Electrical and Computer Engineering University of California San Diego La Jolla CA 92093 USA; ^3^ Department of Mechanical Engineering University of California San Diego La Jolla CA 92093 USA; ^4^ School of Chemistry and Biochemistry and School of Materials Science and Engineering Georgia Institute of Technology Atlanta GA 30332 USA

**Keywords:** interfacial modification, motion detection, organic infrared polymers, retinomorphic sensors

## Abstract

Organic retinomorphic sensors offer the advantage of in‐sensor processing to filter out redundant static backgrounds and are well suited for motion detection. To improve this promising structure, here, the key role of interfacial energetics in promoting charge accumulation to raise the inherent photoresponse of the light‐sensitive capacitor is studied. Specifically, incorporating appropriate interfacial layers around the photoactive layer is crucial to extend the carrier lifetime, as confirmed by intensity‐modulated photovoltage spectroscopy. Compared to its photodiode counterpart, the retinomorphic sensor shows better detectivity and response speed due to the additional insulating layer, which reduces the dark current and the RC time constant. Lastly, three retinomorphic sensors are integrated into a line array to demonstrate the detection of movement speed and direction, showing the potential of retinomorphic designs for efficient motion tracking.

## Graphical abstract

Organic retinomorphic sensors offer the advantage of in‐sensor processing to filter out the redundant static background. To improve this promising structure, incorporating appropriate interfacial layers around the photoactive layer extends the carrier lifetime. Compared to its photodiode counterpart, the retinomorphic sensor shows better detectivity and response speed due to the additional insulating layer to enable efficient motion tracking.

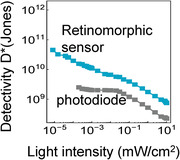

## 1 Introduction

Motion detection is crucial for autonomous navigation, industrial automation, security, and environmental monitoring, but conventional sensors operate at a pre‐determined frame rate and would gather redundant data that slow down signal processing. To enable efficient motion tracking, researchers are seeking an alternative to frame‐based sensing through developing event‐driven devices inspired by biological retina.^[^ ^1^, ^2^, ^3^, ^4^, ^5^, ^6^ ^]^ The retinomorphic photosensors generate pulse signals in response to dynamic changes but not for static illumination. This feature enables in‐sensor computing to remove redundancy and deliver streamlined data upon detecting motion. A class of thin‐film retinomorphic sensors with a capacitor‐resistor design^[^ ^7^, ^8^ ^]^ allows a simpler, higher density implementation than prior silicon‐based circuits^[^ ^9^ ^]^ and makes them well suited for low‐power, high‐speed identification of moving objects, complementary to other bioinspired phototransistor structures.^[^ ^10^, ^11^ ^]^ While a series of insightful papers by Labram^[^ ^12^, ^13^, ^14^, ^15^ ^]^ have discussed device models and basic operating principles, the development of thin‐film retinomorphic devices is nascent and many critical aspects have yet to be explored.

In this work, we show a new approach to increase the detectivity, i.e., signal‐to‐noise ratio, of thin‐film retinomorphic sensors by incorporating interfacial charge‐transporting layers in the photosensitive capacitor. Although charge‐transporting layers are commonly used in conventional photodiodes, they have not been applied to photocapacitors,^[^ ^16^, ^17^ ^]^ perhaps because charge transport is not expected to be a limiting factor in capacitors. However, if we consider the metal‐insulator‐semiconductor (MIS) structure of a photocapacitor, it is foreseeable that the interfaces surrounding the semiconductor could impact charge transfer and recombination, which in turn can be a dominant factor dictating the photoresponse. Therefore this work investigates the effects of interfacial modifications on the performance of retinomorphic detectors, including the dynamic range, detectivity, and response speed. The characteristic recombination time is determined by intensity‐modulated photovoltage spectroscopy which compares the charge dynamics of conventional photodiodes and retinomorphic detectors and reveals the significance of interfacial energetics in device optimization.

While retinomorphic sensors have been implemented using perovskite and organic semiconductors,^[^ ^7^, ^15^ ^]^ previous studies were limited to the visible spectrum. Meanwhile, there is a tremendous need for motion sensing in the near infrared due to various applications in medical imaging, navigation, etc., Leveraging recent advances in organic infrared polymers,^[^ ^18^, ^19^, ^20^, ^21^ ^]^ this study expands the spectral range of retinomorphic detectors to encompass wavelengths spanning from 600 to 1300 nm. In addition to improving individual sensors, we also showcase a demonstration using three retinomorphic pixels for motion tracking up to 11.6 kHz under 940 nm illumination. This proof‐of‐concept result represents a promising advancement toward the scalable integration of organic retinomorphic sensors into detector arrays in the future, to meet the growing demand for more efficient motion‐tracking systems.

## 2 Results and Discussion

**Figure 1:**
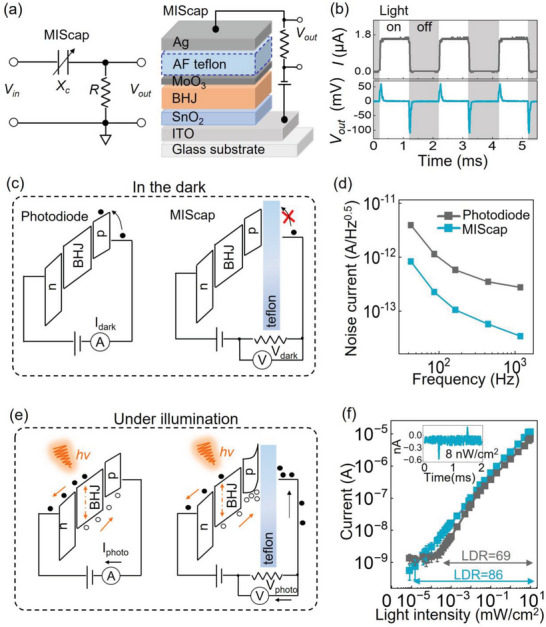
a) A schematic depicting the retinomorphic high‐pass filter circuit and the structure of the capacitive photosensor MIScap. b) Outputs of a conventional photodiode (gray) and a retinomorphic MIScap paired with a 10 kΩ resistor (blue) under 980 nm light of 8 mW cm^−2^. c) Energy band diagrams and d) measurements of noise current versus frequency in the dark. e) Comparison of charge transport and accumulation in a photodiode and in a retinomorphic MIScap. f) Photoresponse versus incident light intensity. The arrows indicate the respective linear dynamic ranges. The inset shows the signal from the retinomorphic sensor under weak light pulses of 8 nW cm^−2^. For all the measurements in this figure, the sensors were at 0 V bias.

The retinomorphic sensor consisted of two components—a photosensitive MIS capacitor (variable reactance *X*_c_) and a resistor (*R*) placed in series, and the two components worked together akin to a resistor‐capacitor (RC) high‐pass filter shown in the circuit schematics of **Figure** **1**a. Below, the term “retinomorphic sensor” refers to the whole combined structure of the MIS capacitor and *R*, while the analysis of the MIS capacitor is focused only on this particular component within the sensor. The photosensitive layer in the MIS capacitor was a bulk heterojunction (BHJ) blend of organic semiconductors sensitive to wavelengths from 600 to 1300 nm.^[^ ^21^, ^22^, ^23^, ^24^ ^]^ The materials of the MIS capacitor and the device fabrication procedure were summarized in the Experimental Section. In comparison to conventional BHJ photodiodes, the MIS capacitor included an additional insulating dielectric. Consequently, when illumination changed, a spike of displacement current was generated in the MIS capacitor; but as the device reached a steady state, the rearrangement of charge stopped and the output returned to zero volts under a static light level. The spiking behavior of the retinomorphic sensor was different from typical photodiodes that maintained a continuous current under constant illumination, as shown in Figure 1b, and this retinomorphic approach to filter out static signals would simplify the subsequent readout circuits for motion tracking.

One might reason that by pairing a conventional photodiode with an external high‐pass filter, the spiking behavior can be emulated. However, an important benefit of using the MIS capacitor over a photodiode is the significant reduction of the dark current noise,^[^ ^25^, ^26^, ^27^, ^28^ ^]^ due to the insulating dielectric suppressing charge injection into the photosensor in Figure 1c. Figure 1d and Figure S1 (Supporting Information) display the dark current measurements, in which the MIS capacitor shows a noise level that is nearly an order of magnitude less than a conventional photodiode.

The working principles of a typical organic photodiode and an MIS capacitor under illumination are presented in Figure 1e. For a photodiode, the photogenerated charge carriers are transported by the electric field and steadily collected at the electrodes. In contrast, the MIS capacitor operates with a transient carrier flow to achieve charge balance across the insulator. On one side (indium tin oxide ITO/tin oxide SnO_2_ in Figure 1e), electrons can exit the semiconductor into the electrode, but on the other side (molybdenum oxide MoO_3_/Teflon), the holes are accumulated at the insulator interface. The accumulated holes are balanced out by an influx of oppositely charged electrons on the Ag electrode next to the insulator. The movement of charges creates a displacement current spike. When the balancing process reaches a steady state, there is no more displacement current flow, and the voltage at the sensor output node returns to the ground. If the illumination is switched off, the change will generate another spike due to the electrons flowing out (a step‐by‐step explanation is provided in Figure S2, Supporting Information, based on Ref.[13]). Thus the retinomorphic sensor sends spiking outputs only when light intensity changes, providing a built‐in feature‐extraction mechanism that is particularly suited to hone in on moving objects.

Regarding the signal amplitude of the retinomorphic sensor, the voltage output *V*_out_ is dependent on the photogenerated displacement current I_P_ induced in the MIS capacitor and the resistor value *R* (*V*_out_ = *I*_p_ *R*). In Figure 1f, the photovoltage of the retinomorphic sensor was converted to current by *I*_p_ = *V*_out_/*R*, to facilitate comparison with the photocurrent of an equivalent photodiode with the same materials stack except for excluding the insulating dielectric. The photodiode signal was indistinguishable from the device background noise when the incident light intensity was below 0.5 µW cm^−2^. Meanwhile, on account of its suppressed noise, the retinomorphic sensor was capable of detecting weak light pulses down to 8 nW cm^−2^ with a signal‐to‐noise ratio >2 as seen in Figure 1f inset and Figure S3 (Supporting Information). The linear dynamic range (LDR) was 69 dB for the photodiode and 86 dB for the retinomorphic sensor. The inherently low noise level in MIS capacitors led to a better detection limit and higher dynamic range than conventional photodiodes.

With respect to increasing the photovoltage *V*_out_ = *I*_p_ *R* of the retinomorphic sensor, it is more challenging to raise the intrinsic photoresponse *I*_p_ of the MIS capacitor compared to adjusting the external load resistance *R* whereby increasing *R* will boost the voltage gain. However, using a higher *R* will incur a trade‐off with the RC delay time and result in a slower response speed. With these considerations in mind, below we identify some key performance bottlenecks and present strategies to optimize the device design.

### 2.1 Improving MIS Capacitor by Tuning Interfacial Energetics

For the MIS capacitor component, the displacement current generated by illumination changes may be limited by charge recombination loss, that when the photogenerated carriers recombine, the electron‐hole pairs are annihilated and cannot contribute to the displacement signal. Reducing the probability of charge recombination is a potential avenue to sustain carrier densities and enhance sensor responsivity.

**Figure 2:**
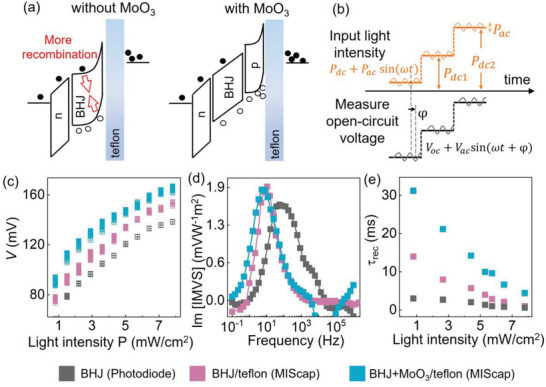
a) Energy band diagrams illustrating the effect of an interfacial p‐layer on charge recombination in the MIScap. b) Probing ac perturbations on device voltage in intensity‐modulated voltage spectroscopy. c) Open‐circuit voltage measured as the light source was at the indicated intensity *P*_dc_ (*x*‐axis values) overlaid with a small perturbation of peak‐to‐peak power *P*_ac_ = 0.16 mW cm^−2^. The same color legend applies to the devices in parts (c–e). The light source was a 980 nm light‐emitting diode. d) Imaginary component of the IMVS transfer function versus the perturbation frequency. The incident light was at *P*_dc_ = 0.78 mW cm^−2^. e) Charge recombination time under various light intensities as in part (c).

We tested the hypothesis that recombination loss can be mitigated by modifying the interfaces adjacent to the BHJ semiconducting layer, as illustrated in **Figure** **2**a. Adding a p‐type MoO_3_ layer between the BHJ and the Teflon dielectric has led to an increase in the peak voltage in the MIS capacitor in Figure S4 (Supporting Information). As the light source was switched on, the output voltage was highest for the MIS capacitor with the MoO_3_ layer, followed by the MIS capacitor without MoO_3_, and then the BHJ photodiode. This trend suggested that the addition of MoO_3_ presented favorable energetics to promote the transfer of photogenerated holes into the p‐layer. The charge transfer reduced the recombination probability and increased the population of accumulated holes next to the Teflon insulator, resulting in a larger photoresponse.

To probe the extent of recombination in different device structures, the charge recombination lifetime was studied using intensity‐modulated photovoltage spectroscopy (IMVS)^[^ ^29^, ^30^, ^31^, ^32^, ^33^ ^]^ in Figure 2b and Figure S4 (Supporting Information). Different from the large‐signal analysis in Figure S5 (Supporting Information), IMVS measured the small perturbations of devices at open‐circuit conditions. The incident light was generated from a sinusoidal waveform with small variations (peak‐to‐peak power *P*_ac_ = 0.16 mW cm^−^^2^) overlaid on a constant level *P*_dc_. The incident light‐induced variations in the device voltage, and the transfer function of voltage output to light input was measured by a potentiostat. Figure 2c shows the open‐circuit voltage measured at different light intensities. Since the measurements were carried out at open‐circuit states^[^ ^34^ ^]^ without current flow, IMVS excluded complexities from transport and isolated recombination to be the main mechanism affecting the carrier population. Thus the frequency characteristics of IMVS data would enable the inference of carrier recombination lifetime.

Figure 2d provides examples of IMVS impedance versus the perturbation frequency of the input light. The imaginary component of the IMVS impedance reached a peak at a characteristic frequency *f*_c_, and this frequency corresponded to the carrier recombination lifetime *τ*_rec_ = 1/(2π*f*_c_). The characteristic frequency was the lowest for the MIS capacitor with the MoO_3_ interfacial layer, followed by the MIS capacitor without MoO_3_, and then the conventional photodiode. Consequently, the recombination lifetime followed the order of MIS capacitor with MoO_3_ > MIS capacitor without MoO_3_ > photodiode, as shown in Figure 2e and Figure S4b (Supporting Information) for a range of incident light power. The conventional photodiode showed the shortest recombination lifetime probably due to accessible charge transfer into the electrodes, while the MIS capacitors had one electrode blocked by the insulator and retained the accumulated charges until they recombined. The recombination lifetime was at least two times longer in the MIS capacitor with the MoO_3_ interface than the device without it, verifying that recombination in the BHJ was suppressed as charges moved into interfacial layers. This interfacial modification increased the intrinsic photo‐signal in the MIS capacitor, different from the extrinsic gain mechanism of sizing up the resistor of the retinomorphic sensor, to be discussed in the next section.

### 2.2 Optimizing Load Resistance and Operating Conditions

**Figure 3:**
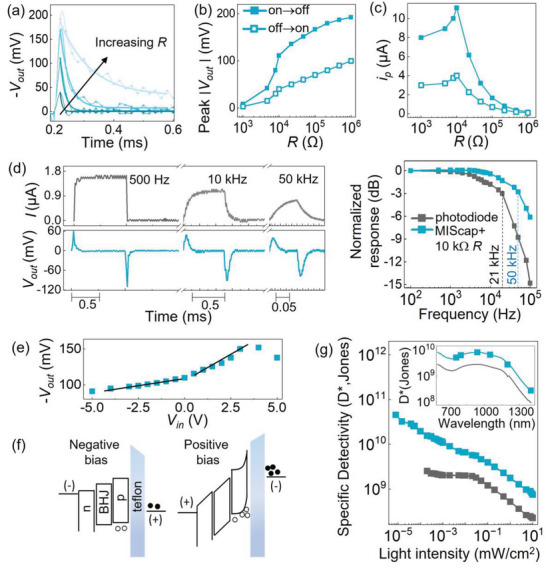
a) Voltage output of the retinomorphic sensor with different resistors ranging from 1 kΩ to 1 mΩ. The signal for 1 kΩ was multiplied by 5 for ease of comparison. The sensor MIScap included the p‐MoO_3_ interfacial layer. A change in light power of Δ*P* = 8 mW cm^−^^2^ from a 980 nm LED was used in all of the following measurements unless indicated otherwise. b,c) The sensors were at 0 V bias unless noted otherwise Peak voltage and transient photocurrent (*i*_p_) of the retinomorphic sensor as a function of the resistor value, as the light source was switching on→off or off→on. d) Signal magnitude versus light modulation frequency. Gray: photodiode; blue: retinomorphic sensor. The resistor in the retinomorphic detector was 10 kΩ for the measurements in parts (d–g). e) Peak voltage of the retinomorphic sensor versus applied bias voltage. The black lines are fitted to Equation 1. f) Energy band diagrams comparing the extent of charge accumulation under different applied biases. g) Specific detectivity versus incident light intensity. The inset shows the detectivity versus light wavelength at Δ*P* = 0.08 mW cm^−^^2^ modulated at 500 Hz for measurements on the retinomorphic sensor and 313 Hz for the photodiode.

In this work, the retinomorphic sensor was a combination of a photo‐sensing MIS capacitor and a load resistor that influences the voltage gain as *V*_out_ = *I*_p_ *R*. In **Figure** **3**a–c, we used the same MIS capacitor with MoO_3_ and paired it with different resistors ranging in values from 1 kΩ to 1 mΩ. Increasing the resistance led to higher voltage outputs, but the RC time constant was also prolonged in Figure 3a.

The peak output voltage as a function of the load resistance in Figure 3b shows that the output rose from a few mV at 1 kΩ to nearly 200 mV at 1 mΩ. The signal induced by the incident light being switched off (on→off) was consistently higher than when the light was switched on (off→on). This finding is similar to the results observed by Labram,^[^ ^13^ ^]^ and we speculate that the charge rearrangement from on→off state involves quick recombination and a rush of outflowing carriers to rebalance the charge distribution across the insulator. On the other hand, the off→on condition involves slower processes to generate and accumulate carriers at the interface, and the displacement current flow would be less than the on→off scenario.

The data in Figure 3b are presented from another perspective by plotting the peak transient current *i*_p_ = peak *V*_out_/*R* versus the load resistance in Figure 3c. The *i*_p_ was the highest around the resistance of 10 kΩ. This plot brought into focus that although the voltage output increased with *R*, the rate of increase plateaued with large *R* values. For *R* greater than tens of kΩ, the device has reached the point of diminishing return for voltage gain, and large *R* values would also delay the RC time constant and gain‐bandwidth product.^[^ ^35^ ^]^ Hence, in the following characterization, the retinomorphic sensor was set with a 10 kΩ resistor to attain sufficient voltage gain and retain fast response speed.

Figure 3d presents the temporal response of the retinomorphic sensor and its photodiode counterpart under a 980 nm light‐emitting diode (LED) modulated up to 100 kHz. As the light switching frequency increased, the retinomorphic sensor showed less attenuation in its output amplitude than the photodiode. The −3 dB cut‐off frequency was 50 kHz for the retinomorphic sensor and 21 kHz for the photodiode. The mechanism of displacement current and charge rearrangement in the retinomorphic sensor imposed less stringent requirements on charge transport, unlike in conventional photodiodes where charges must be transported to be collected as the photocurrent. The retinomorphic sensor also included the insulating dielectric in series with the semiconductor, which reduced the capacitance and RC time thus accelerated its response speed.

The applied bias *V*_in_ on the retinomorphic sensor was another tunable parameter affecting the peak output voltage *V*_out_ in Figure 3e. With a more positive *V*_in_, the *V*_out_ gradually increased until the applied bias was >3.5 V and saturated at higher voltage, possibly because a higher electric field no long assisted charge dissociation. The difference in *V*_out_ was only 50 mV for a large range of applied bias from −5 to 3.5 V. This small change indicated that the applied bias was mostly dropped across the insulating dielectric and thus induced only a slight shift in the energy levels of semiconducting layers as illustrated in Figure 3f. Nonetheless, a positive bias made it favorable for holes to accumulate at the MoO_3_/Teflon interface and in turn facilitated the rise in *V*_out_. There was a change in slope ≈0 V bias in Figure 3e; the linear‐fit slopes were 0.011 for the region of *V*_in_ from 0 to 3.5 V and 0.005 for the region of *V*_in_ from −5 to 0 V. Using the model of a voltage divider, the slope corresponds to the impedance ratio in the following expression:

(1) $$\;V_{\mathrm{out}}=\frac{R}{X_{c}+R}\;V_{\mathrm{in}},\;\;\;X_{c}=\frac{1}{2\pi fC}$$

where *X*_c_ is the MIS capacitive reactance (Ω), *f* is the modulation frequency (Hz), and *C* is the capacitance (F). Setting the slope fit values to *R*/(*X*_c_+*R*) and with the known values of *R* = 10 kΩ and *f* = 500 Hz, the extrapolated capacitance ranges from 0.16 to 0.35 nF. We note that this model is very simplified, but this level of change due to light modulation is reasonable for the MIS capacitor with a geometric capacitance measured to be ≈2 nF.

Previously Figure 1f inset and Figure S3 (Supporting Information) have shown the detection limit of the MIS capacitor with MoO_3_ layer, for which an input light intensity of 8 nW cm^−^^2^ yielded a signal‐to‐noise ratio of 3. Then, given the detector area of 0.09 cm^2^, the equivalent noise power^[^ ^36^ ^]^ was *NEP* = 0.24 nW (=8/3 nW cm^−^^2^ × 0.09 cm2) for the retinomorphic detector with 10 kΩ resistor and under zero bias. For ease of comparison with other detector papers, Figure 3g provides the detectivity versus the incident light intensity, as well as the detectivity versus the spectral wavelength in the inset. Our devices were responsive to infrared spectral wavelengths from 700 to 1300 nm. The detectivity reached 3 × 1010 Jones for the retinomorphic sensor and 2 × 109 Jones for the photodiode. The detectivity was relatively constant for the photodiode at low light levels, but for the MIS capacitor, the detectivity increased with decreasing light intensity, because of the tendency that recombination abated at low charge density.^[^ ^37^ ^]^

### 2.3 Characterizing the Retinomorphic Pixels in a Line Array

**Figure 4:**
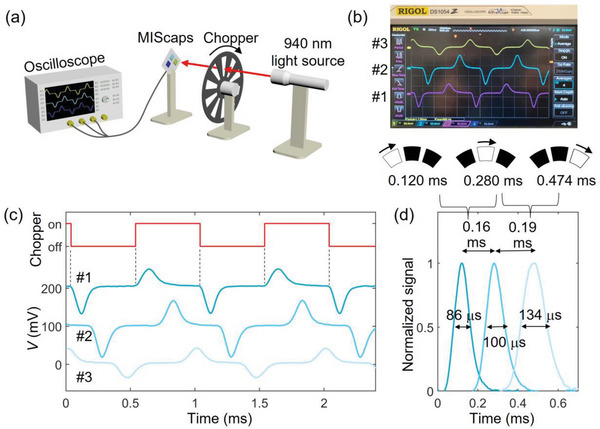
a) A measurement setup using three retinomorphic pixels to detect motion signals modulated by the light chopper wheel. b) A screenshot of the signals captured by an oscilloscope. Below the screenshot, the diagram shows the points in time when an edge of the chopper blade moved across the pixels. c) Light pulses modulated by the chopper blade (red) and the corresponding signals measured by the retinomorphic pixels (blue). The curves are offset by 100 mV for viewing clarity. The chopper pulse waveform was lined up with the first pixel, and with rotation the chopper edge moved over the second and the third pixel. d) Normalized signals of the three pixels as a function of the recording time. The time intervals of FWHM are marked on each curve to indicate the temporal resolution for detecting edge movement.

After clarifying the operating principles of individual retinomorphic sensors, our next step was to place them in an array and show the feasibility to detect light movement across lateral space. In **Figure** **4**a, we set up three retinomorphic sensors lined up over an arc corresponding to 7° between pixel locations (Figure S6, Supporting Information). A 940 nm LED light beam was modulated by a mechanical chopper wheel rotating at 1 kHz, such that the light edge moved at 36° m^−1^s^−1^ (=360°/10 because the chopper blade had 10 equally spaced window slots) over the line of sensors. The signals of the three retinomorphic pixels were simultaneously captured by an oscilloscope, as shown in Figure 4b. The measurement showed sequential voltage spikes as each pixel detected the light movement over its sensor area in Figure 4c. For ease of comparison, Figure 4d presents a set of normalized peaks as the light edge passed over the sensor pixels. The time difference between the voltage spikes allowed us to extract the lateral movement speed of the chopper blade edge. The time intervals were 0.16 ms between the peaks of pixels #1 and #2 (0.12 and 0.28 ms, respectively), and 0.19 ms between pixels #2 and #3 (0.28 and 0.474 ms, respectively). From the known spacing of 7° between pixels, the calculated speed was 36—44° m^−1^s^−1^, in good agreement with the known chopper rotation speed of 36° m^−1^s^−1^. The uncertainty in the speed calculation may be attributed to light scattering or slight misalignment between the sensor arc and the chopper light beam arc. Furthermore, the full‐width at half‐maximum (FWHM) of the spiking signals ranged between 86 and 134 µs. The FWHM intervals represented the temporal resolution of the detectors, indicating that edge motion up to 11.6 kHz (=1/86 µs) would be resolvable without ghost edges at pixel #1. The spatiotemporal resolution might be improved in the future by reducing the sensor area since a smaller area decreases the MIS capacitance to shorten the rise and fall times and enables a higher density of pixels.

## 3 Conclusion

For the next generation of low‐power high‐speed motion detectors, organic thin‐film retinomorphic sensors emerged as a promising candidate to offer the advantages of in‐sensor processing to filter out redundant static backgrounds and hone in on moving features. The spiking mechanism was implemented with a device structure that was simple to fabricate and scalable to high density. Moreover, the device's spectral response would be easily adjustable from visible to infrared by tuning the organic semiconductors.

From a perspective of comparing the materials and design choices for retinomorphic sensors and photodiodes, this study focused on the interfacial layers next to the BHJ layer, to clarify the role of charge‐transporting layers in retinomorphic sensors. Leveraging the IMVS technique commonly used in photovoltaic research to determine carrier recombination lifetimes, we found that incorporating appropriate interfacial layers around the BHJ layer was crucial to extend the carrier lifetime. Specifically, the interfacial layer of MoO_3_ reduced recombination by at least twofold and significantly enhanced the inherent charge accumulation and rearrangement within the photosensitive MIS capacitor.

Having improved the MIS capacitor component, we studied the effect of the resistor component on the retinomorphic sensor output. The *V*_out_/*R* ratio was used to select an optimized resistor value that balances the voltage gain and response speed. The retinomorphic sensor with a 10 kΩ resistor showed a −3 dB frequency of 50 kHz and a detectivity of 3 × 1010 Jones, achieving better performance than its photodiode counterpart. The reason for the high performance was that the insulator layer in the MIS capacitor helped to reduce dark current and also lower the capacitance and in turn the RC time constant.

Lastly, three retinomorphic sensors were integrated into a line array to demonstrate the detection of movement speed and direction, which required simultaneous measurements by multiple pixels to extract that information. The proof‐of‐concept demonstration was able to resolve rotation up to 11.6 kHz. In summary, this work has offered key design guidelines to improve the exciting retinomorphic technology. While there are questions remaining such as the asymmetric response^[^ ^12^ ^]^ of the sensor during switching, it may be a powerful combination in the future to integrate organic retinomorphic sensors with a neuromorphic processor, to finally meet the requirements for implementing real‐time machine learning in navigation and imaging diagnostic applications.

## 4 Experimental Section

### Materials Preparation

The narrow bandgap polymer (poly(4‐(5‐(4‐(3,5‐bis(dodecyloxy)‐benzylidene)−4*H*‐yclopenta[2,1‐*b*:3,4‐*b* *'*]dithiophen‐2‐yl)thiophen‐2‐yl)−6,7‐dioctyl‐9‐(thiophen‐2‐yl)‐[1,2,5]thiadiazolo[3,4‐*g*]quinoxaline)) (CDT‐TQ) was synthesized as discussed in Ref.[22] The acceptor [6,6]‐phenyl‐C71‐butyric acid methyl ester (PC_70_BM) was purchased from Ossila Ltd. The Teflon solution was purchased from Chemours (product# AF 2400). The materials were used as received. The CDT‐TQ, PC_70_BM, and camphoric acid (Aldrich) were blended at 2:4:1 weight ratio dissolved in 1,2‐dichlorobenzene at a concentration of 28 mg mL^−1^. An additive 1,8‐diiodooctane was added to the blend solution at a volume ratio of 3%. This BHJ blend solution was stirred at 70 °C for at least 24 h in the glovebox. The precursor solution of SnO_2_ was prepared by dissolving SnCl_2_.H_2_O in ethanol at a concentration of 0.07 m and then stirred in a water bath at 80 °C for 8 h.^[^ ^23^ ^]^

### Device Fabrication

ITO substrates were cleaned with detergent, deionized water, and sonication in isopropanol and acetone. The electron‐transport layer SnO_2_ was prepared by spin‐coating the precursor solution at 3000 rpm for 40 s, followed by annealing at 180 °C for 1 h with a thickness ≈of 20–40 nm. The substrates were then transferred to a nitrogen‐filled glovebox for the deposition of other layers. The BHJ blend solution was spin‐coated at 2000 rpm for 40 s on top of the SnO_2_ layer to form a film with a thickness of 200 nm. Then 10 nm MoO_3_ was deposited by thermal evaporation. Afterward, for the MIS capacitor structure, the Teflon solution was spin‐coated on top of the BHJ layer at 1500 rpm for 40 s three times (≈750 nm thickness) and left to stand overnight. A 100 nm Ag layer was thermally deposited through a shadow mask, defining an active area of 0.09 cm^2^. For the photodiode structure, the device consisted of ITO/SnO_2_/BHJ/MoO_3_/Ag, with the same layer thicknesses as the MIS capacitor for a fair comparison. The energy diagram and materials structure are summarized in Figure S7 (Supporting Information). All the devices were encapsulated by glass cover slides and characterized in ambient conditions.

### Device Characterization

The current–voltage characteristics were measured through a source meter (Keithley 2400) controlled by custom Labview software. The light was incident from the transparent ITO side through the glass substrate. The magnitude and modulation frequency of the incident light sources were adjusted by a function generator (Rigol, DG 2401A), using different LEDs with peak wavelengths of 760, 800, 980, 1050, 1200, and 1550 nm (from Thorlabs). The light intensities were calibrated with a germanium detector (Newport, 818IR). Besides the LED light sources, for Figure 3g, another monochromatic light source was used for measuring the photodiode characteristics. The photodiode response was amplified through a pre‐amplifier (Stanford Research Systems, SRS 570) and recorded by a lock‐in amplifier (Stanford Research Systems, SRS 530). For measurements of MIS capacitors, the *V*_out_ across the external load resistor R was recorded through an oscilloscope (Rigol, DS1054), while *V*_in_ was controlled by the Keithley source meter. The noise spectral densities were measured in the dark, where the device was connected to the SRS 570 pre‐amplifier and then to the SRS 530 lock‐in amplifier. The reference frequency in the noise current measurements was controlled by a function generator serving as the external trigger for the lock‐in amplifier. The device noise was calculated by subtracting the equipment background noise. IMVS measurements were carried out by using a potentiostat (SP‐200 from BioLogic) and the 980 nm LED light source.

### Imaging of the Rotating Optical Chopper

Three MIS capacitors, each 3 × 3 mm^2^, on the same glass substrate were fabricated. The center‐to‐center distance between two adjacent MIS capacitors was 7 mm, which corresponded to ≈7° rotation angle of the chopper blade. Each MIS capacitor was connected to a 47 kΩ resistor, and the three devices were connected to three different channels of an oscilloscope (Rigol, DS1054) respectively. A 940 nm focused infrared light torch (Bestsun, IR‐940) was used as the light source, and the light spot was ≈2 cm in diameter. The light was modulated by a chopper (Thorlabs, MC2000) with a 10‐slot blade (Thorlabs, MC1F10) at a 1 kHz chopping frequency. Some 3D‐printed holders from a customized Raise 3D printer^[^ ^38^, ^39^ ^]^ were used to align the light source, chopper, and the arc on which the sensor pixels were located.

### Photo‐Response Equations

The device responsivity *ℜ* was extracted from the measured photocurrent or converted from the *V*_out_ signal:

(2) $$\;{ℜ}_{\mathrm{photodiode}}=\frac{\left(I_{p}-I_{\mathrm{dark}}\right)}{\Delta P}\;,\;\;{ℜ}_{\mathrm{MISCap}}=\frac{\left(\frac{V_{\mathrm{out}}}{R}\right)}{\Delta P}\;$$

where *I*_p_ is the photocurrent under illumination, *I*_dark_ is the current in the dark, Δ*P* is the intensity of the incident light, and *R* is the external resistor connected to the MIS capacitor. The specific detectivity (D*) was defined by:

(3) $$\;D^{*}=\frac{\sqrt{A\Delta f}}{NEP}\;=\frac{ℜ\sqrt{A}}{S_{n}}\;$$

where *ℜ* is the spectral responsivity, *A* is the active area in the device, Δ*f* is the measurement bandwidth, NEP is the noise equivalent power, and *S*_n_ is the noise spectral density in current per square root hertz.

## Conflict of Interest

The authors declare no conflict of interest.

## Author Contributions

S.W., L.Z., Y.Z., and C.S. carried out the experimental fabrication, measurements, and analysis. N.E. and J.D.A. provided the organic infrared semiconductors. T.N.N. conceptualized the device mechanistic studies and supervised the project. All authors contributed to the writing and editing of this manuscript. S.‐E.W., L.Z., and Y.Z. contributed equally to this work.

## Supporting information

Supporting Information

## Data Availability

The data that support the findings of this study are available from the corresponding author upon reasonable request.

## References

[1] C. Posch , T. Serrano‐Gotarredona , B. Linares‐Barranco , T. Delbruck , Proceedings of the IEEE , IEEE, NY, USA 2014, pp. 1470–1484.

[2] Z. Zhang , S. Wang , C. Liu , R. Xie , W. Hu , P. Zhou , Nat. Nanotechnol. 2022, 17, 27. 34750561 10.1038/s41565-021-01003-1

[3] H. Chen , L. Lv , Y. Wei , T. Liu , S. Wang , Q. Shi , H. Huang , Cell Rep. Phys. Sci. 2021, 2, 100507.

[4] H. Wang , Q. Zhao , Z. Ni , Q. Li , H. Liu , Y. Yang , L. Wang , Y. Ran , Y. Guo , W. Hu , Y. Liu , Adv. Mater. 2018, 30, 1803961. 10.1002/adma.201803961 30252955

[5] H. Jang , C. Liu , H. Hinton , M. H. Lee , H. Kim , M. Seol , H. J. Shin , S. Park , D. Ham , Adv. Mater. 2020, 32, 2002431. 10.1002/adma.202002431 32700395

[6] S. J. Kim , J. S. Jeong , H. W. Jang , H. Yi , H. Yang , H. Ju , J. A. Lim , Adv. Mater. 2021, 33, 2100475. 10.1002/adma.202100475 34028897

[7] C. Trujillo Herrera , J. G. Labram , Appl. Phys. Lett. 2020, 117, 233501.

[8] L. Reissig , S. Dalgleish , K. Awaga , AIP Adv. 2016, 6, 015306.

[9] F. Liao , F. Zhou , Y. Chai , J. Semiconductors 2021, 42, 013105.

[10] B. Shao , T. Wan , F. Liao , B. J. Kim , J. Chen , J. Guo , S. Ma , J. H. Ahn , Y. Chai , ACS Nano 2023, 17, 10291. 37186522 10.1021/acsnano.3c00487

[11] J. Chen , Z. Zhou , B. J. Kim , Y. Zhou , Z. Wang , T. Wan , J. Yan , J. Kang , J. H. Ahn , Y. Chai , Nat. Nanotechnol. 2023, 10.1038/s41565-023-01379-2. 37081081

[12] J. G. Labram , J. Phys. D Appl. Phys. 2023, 56, 065105.

[13] X. Zhang , J. G. Labram , J. Mater. Chem. C Mater. 2022, 10, 12998.

[14] C. Trujillo Herrera , J. G. Labram , J. Phys. D Appl. Phys. 2021, 54, 475110.

[15] C. Trujillo Herrera , J. G. Labram , ACS Appl. Electron. Mater. 2022, 4, 92.

[16] T. Saxena , M. Shur , IEEE Trans. Electron Devices 2016, 63, 3236.

[17] D. Caputo , G. De Cesare , A. Nascetti , F. Palma , M. Petri , Appl. Phys. Lett. 1998, 72, 1229.

[18] N. Li , P. Mahalingam , J. H. Vella , D.‐S. S. Leem , J. D. Azoulay , T. N. Ng , Mater. Sci. Eng. R‐Rep. 2021, 146, 100643.

[19] Q. He , A. Basu , H. Cha , M. Daboczi , J. Panidi , L. Tan , X. Hu , C. C. Huang , B. Ding , A. J. P. White , J. S. Kim , J. R. Durrant , T. D. Anthopoulos , M. Heeney , Adv. Mater. 2023, 35, 2209800. 10.1002/adma.202209800 36565038

[20] P. Jacoutot , A. D. Scaccabarozzi , T. Zhang , Z. Qiao , F. Aniés , M. Neophytou , H. Bristow , R. Kumar , M. Moser , A. D. Nega , A. Schiza , A. Dimitrakopoulou‐Strauss , V. G. Gregoriou , T. D. Anthopoulos , M. Heeney , I. McCulloch , A. A. Bakulin , C. L. Chochos , N. Gasparini , Small 2022, 18, 2200580. 10.1002/smll.202200580 35246948

[21] J. H. Vella , L. Huang , N. Eedugurala , K. Mayer , T. N. Ng , J. Azoulay , Sci. Adv. 2021, 7, abg2418. 10.1126/sciadv.abg2418 PMC8189577 34108215

[22] Z. Wu , Y. Zhai , W. Yao , N. Eedugurala , S. Zhang , L. Huang , X. Gu , J. D. Azoulay , T. N. Ng , Adv. Funct. Mater. 2018, 28, 1805738.

[23] C. Shin , N. Li , B. Seo , N. Eedugurala , J. D. Azoulay , T. N. Ng , Mater. Horiz. 2022, 9, 2172. 35642962 10.1039/d2mh00479h

[24] N. Li , N. Eedugurala , D. S. Leem , J. D. Azoulay , T. N. Ng , Adv. Funct. Mater. 2021, 31, 2100565.

[25] R. Ollearo , J. Wang , M. J. Dyson , C. H. L. Weijtens , M. Fattori , B. T. van Gorkom , A. J. J. M. van Breemen , S. C. J. Meskers , R. A. J. Janssen , G. H. Gelinck , Nat. Commun. 2021, 12, 7277. 34907190 10.1038/s41467-021-27565-1 PMC8671406

[26] J. Kublitski , A. Hofacker , B. K. Boroujeni , J. Benduhn , V. C. Nikolis , C. Kaiser , D. Spoltore , H. Kleemann , A. Fischer , F. Ellinger , K. Vandewal , K. Leo , Nat. Commun. 2021, 12, 551. 33483507 10.1038/s41467-020-20856-z PMC7822930

[27] Z. Wu , N. Li , N. Eedugurala , J. D. Azoulay , D.‐S. Leem , T. N. Ng , npj Flex. Electronics 2020, 4, 6.

[28] S. Gielen , C. Kaiser , F. Verstraeten , J. Kublitski , J. Benduhn , D. Spoltore , P. Verstappen , W. Maes , P. Meredith , A. Armin , K. Vandewal , Adv. Mater. 2020, 32, 2003818. 10.1002/adma.202003818 33078513

[29] J. Halme , Phys. Chem. Chem. Phys. 2011, 13, 12435. 21655592 10.1039/c1cp21134j

[30] E. Guillén , F. J. Ramos , J. A. Anta , S. Ahmad , J. Phys. Chem. C 2014, 118, 22913.

[31] Y. T. Set , B. Li , F. J. Lim , E. Birgersson , J. Luther , Appl. Phys. Lett. 2015, 107, 173301.

[32] A. Bou , A. Pockett , D. Raptis , T. Watson , M. J. Carnie , J. Bisquert , J. Phys. Chem. Lett. 2020, 11, 8654. 32955259 10.1021/acs.jpclett.0c02459

[33] J. Bisquert , M. Janssen , J. Phys. Chem. Lett. 2021, 12, 7964. 34388001 10.1021/acs.jpclett.1c02065 PMC8404195

[34] M. Kielar , T. Hamid , M. Wiemer , F. Windels , L. Hirsch , P. Sah , A. K. Pandey , Adv. Funct. Mater. 2020, 30, 1907964.

[35] Y. Li , G. Chen , S. Zhao , C. Liu , N. Zhao , Sci. Adv. 2022, 8, eabq0187. 36149950 10.1126/sciadv.abq0187 PMC9506725

[36] Y. Fang , A. Armin , P. Meredith , J. Huang , Nat. Photonics 2019, 13, 1.

[37] H. Kim , Z. Wu , N. Eedugurala , J. D. Azoulay , T. N. Ng , ACS Appl. Mater. Interfaces 2019, 11, 36880. 31524369 10.1021/acsami.9b08622

[38] Y. Zhai , Z. Wang , K. S. Kwon , S. Cai , D. Lipomi , T. N. Ng , Adv. Mater. 2020, 33, 2002541. 10.1002/adma.202002541 33135205

[39] S. E. Wu , N. Phongphaew , Y. Zhai , L. Yao , H. H. Hsu , A. Shiller , J. D. Azoulay , T. N. Ng , Nanoscale 2022, 14, 16110. 36281764 10.1039/d2nr04382c

