# Supplementary material for: Retinomorphic Motion Detector Fabricated with Organic Infrared Semiconductors
Source: Adv Sci (Weinh). 2023 Sep 6;10(31):2304688. doi: 10.1002/advs.202304688 (PMC10625071; doi:10.1002/advs.202304688)
Supplement: Supplementary file 1 — Supporting Information [file ADVS-10-2304688-s001.pdf]

ADVANCED SCIENCE
Open Access

Supporting Information

for *Adv. Sci.*, DOI 10.1002/advs.202304688

Retinomorphic Motion Detector Fabricated with Organic Infrared Semiconductors

*Shuo-En Wu, Longhui Zeng, Yichen Zhai, Chanho Shin, Naresh Eedugurala, Jason D. Azoulay and Tse Nga Ng**

Supporting Information

Retinomorphic Motion Detector Fabricated with Organic Infrared Semiconductors

Shuo-En Wu[1+], Longhui Zeng[2+], Yichen Zhai[3+], Chanho Shin[1], Naresh Eedugurala[4], Jason D. Azoulay[4], Tse Nga Ng[1,2*]

[1] Materials Science and Engineering Program, University of California San Diego, La Jolla, CA 92093, United States.

[2] Department of Electrical and Computer Engineering, University of California San Diego, La Jolla, CA 92093, United States.

[3] Department of Mechanical Engineering, University of California San Diego, La Jolla, CA 92093, United States.

[4] School of Chemistry and Biochemistry, Georgia Institute of Technology, Atlanta, GA 30332, United States.

+Equal contribution

*Corresponding email: tnn046@ucsd.edu

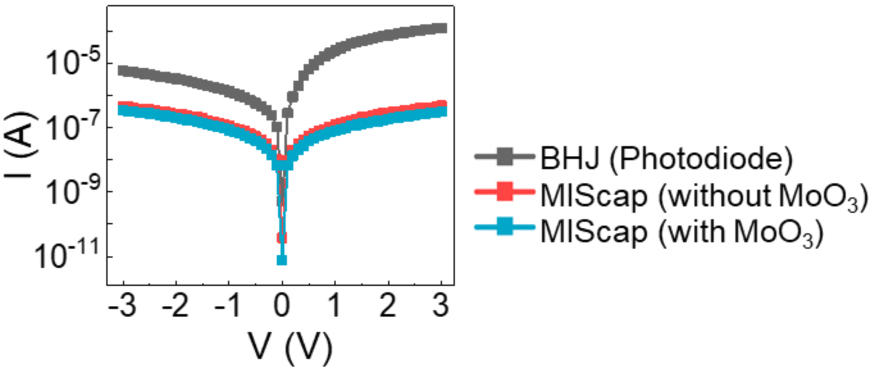

Figure S1. Current-voltage characteristics in the dark.

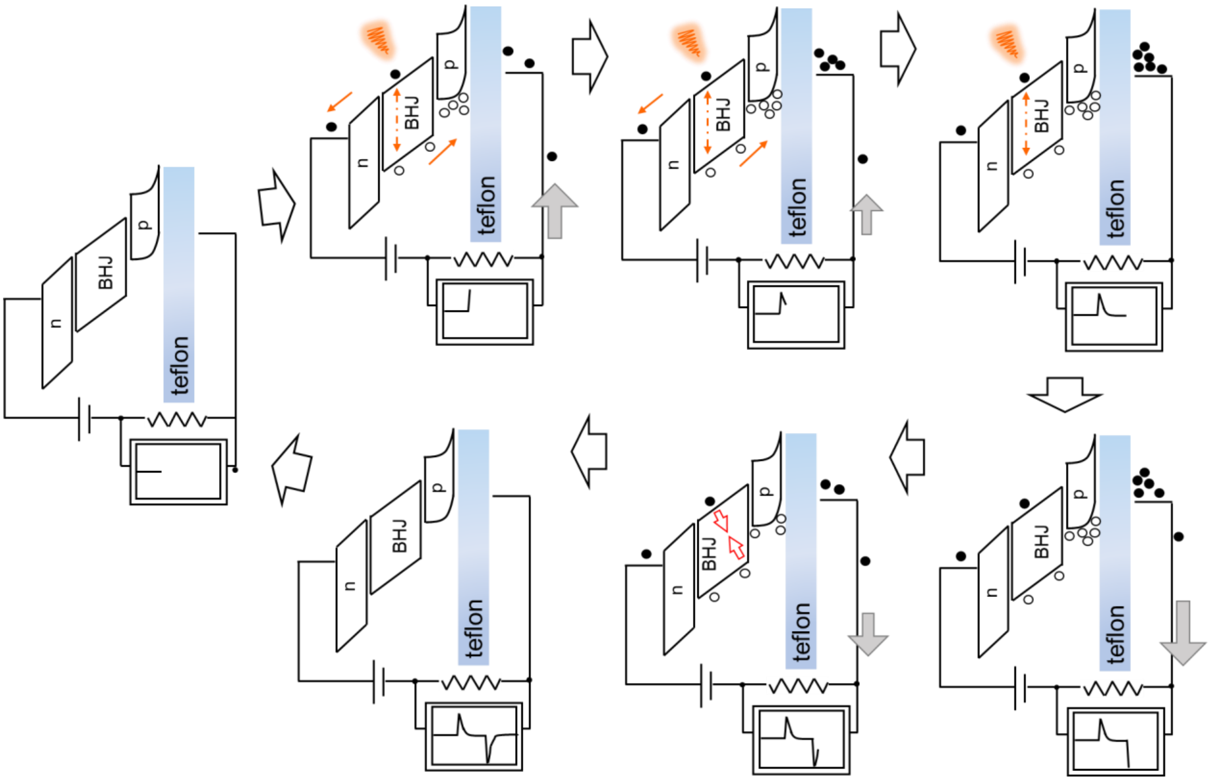

Figure S2. Schematics on charge dynamics in the retinomorphic sensor during different phases of light illumination. The gray arrows indicate the direction and the magnitude of electrons flowing into or out of the Ag electrode on the right side of the teflon insulator. The oscilloscope trace indicates the voltage response versus time when light is switched on (top row) and switched off (bottom row). This diagram is based on the paper by Trujillo Herrera, C.; Labram, J. G. An Organic Retinomorphic Sensor. *ACS Appl Electron Mater* **2022**, *4* (1), 92–98.

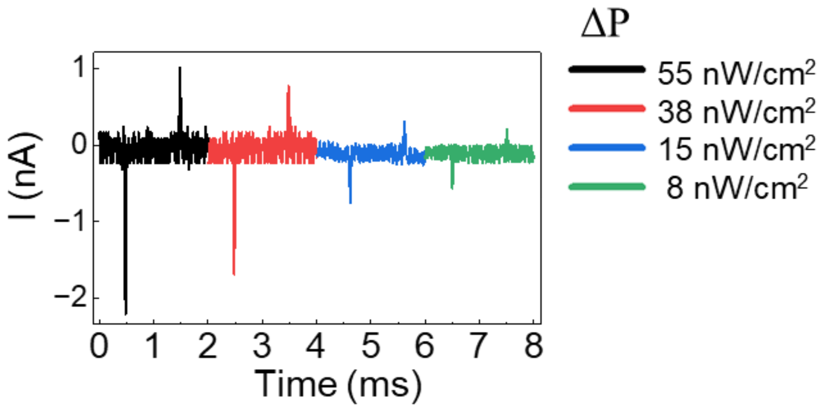

Figure S3. Voltage response of the retinomorphic sensor as the incident light power was changed from 55-8 nW/cm^2. The data showed the noise equivalent power of our device.

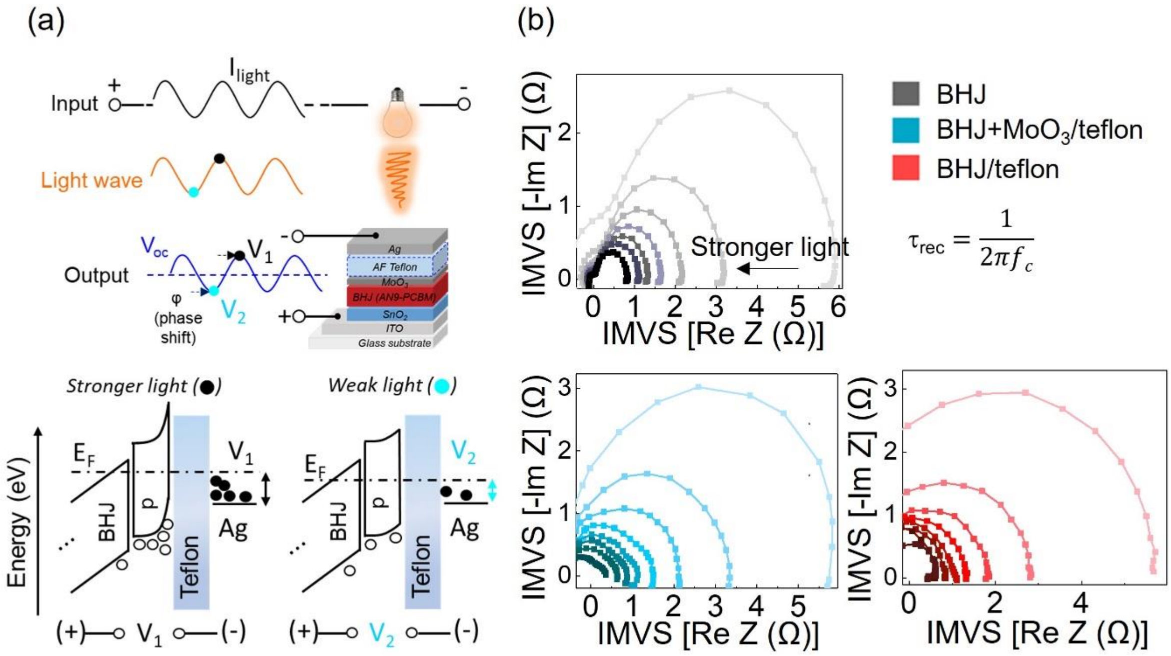

Figure S4. Schematics and data of Intensity Modulated Photovoltage Spectroscopy (IMVS). (a) Schematics of input light perturbation with respect to the measurement of open-circuit voltage in the IMVS technique. (b) Nyquist plots of real vs imaginary impedance as a function of light intensity for the different devices tested in this work.

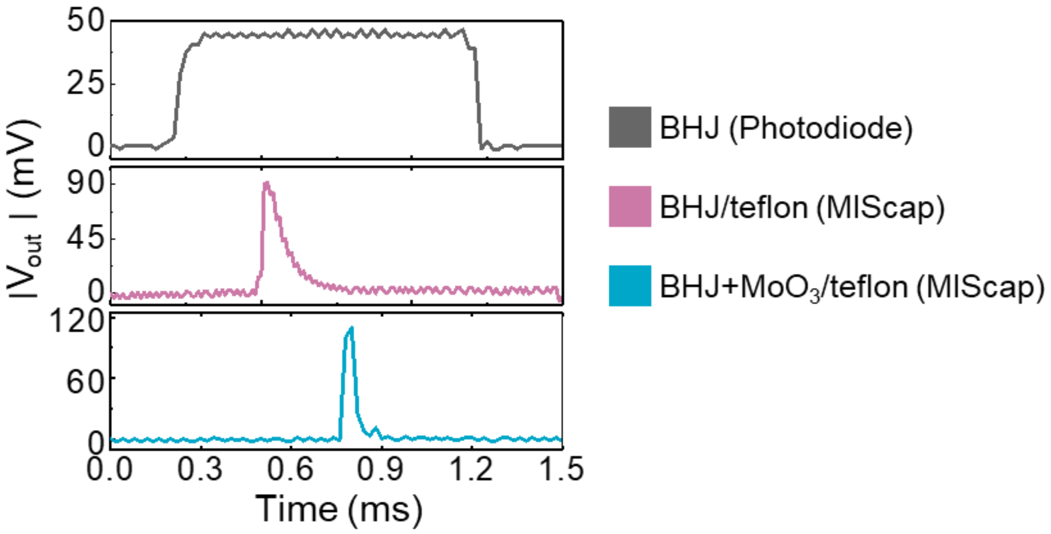

Figure S5. Output voltage versus time under a light pulse at 500 Hz to compare rise/fall time of different devices.

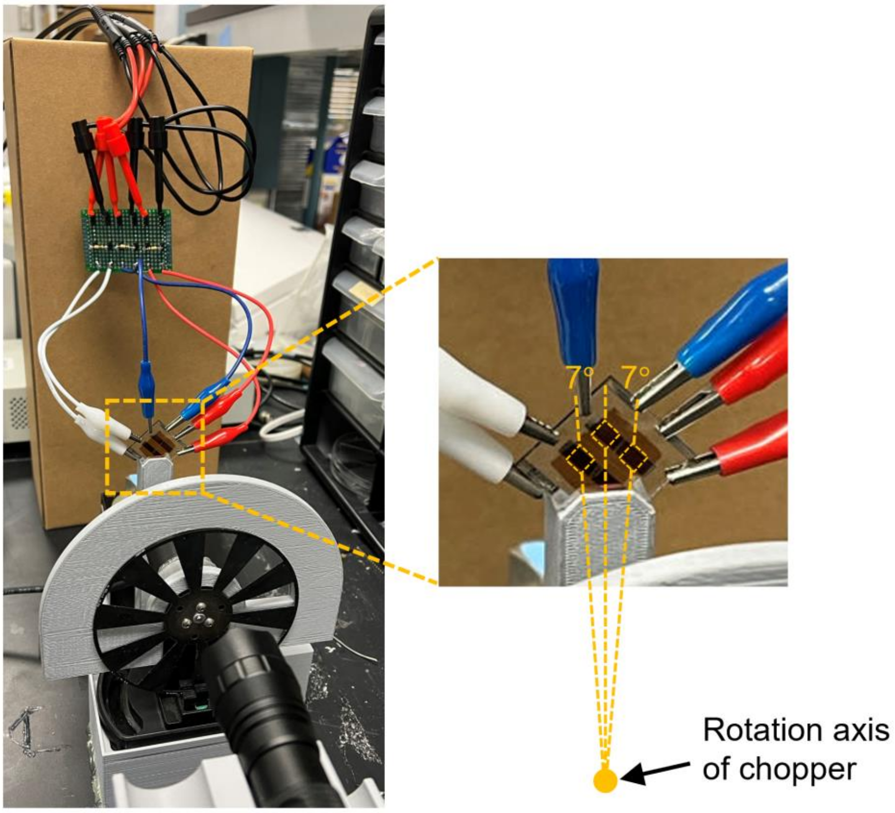

Figure S6. Photographs of the three retinomorphic sensors placed on an arc. The spacings between individual retinomorphic pixels were approximately 7 degrees with respect to the rotation axis of the mechanical chopper.

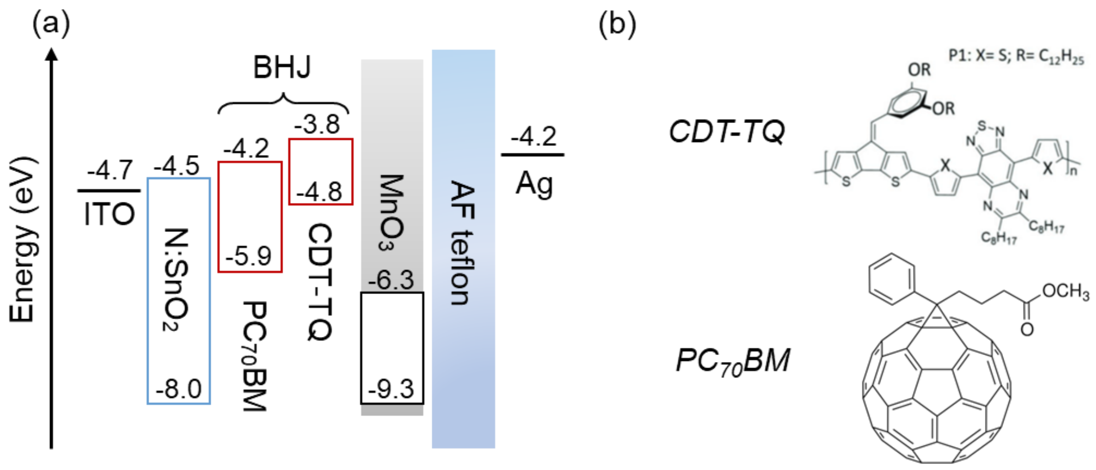

Figure S7. (a) Energy diagram for the materials used in this work. (b) Molecular structure of the donor (CDT-TQ) and acceptor ($PC_{70}BM$) in the BHJ layer.
